# Editorial: Role of Ion Channels in Pain

**DOI:** 10.3389/fphar.2022.884665

**Published:** 2022-06-06

**Authors:** Jinwei Zhang, Jing Yao, Mingqiang Rong

**Affiliations:** ^1^ State Key Laboratory of Bioorganic and Natural Products Chemistry, Research Center of Chemical Kinomics, Shanghai Institute of Organic Chemistry, Chinese Academy of Sciences, Shanghai, China; ^2^ School of Medicine, Institute of Cardiovascular Diseases, Xiamen Cardiovascular Hospital Xiamen University, Xiamen University, Xiamen, China; ^3^ Hatherly Laboratories, College of Medicine and Health, Institute of Biomedical and Clinical Sciences, University of Exeter Medical School, Streatham Campus, Exeter, United Kingdom; ^4^ State Key Laboratory of Virology, Frontier Science Center for Immunology and Metabolism, Department of Anesthesiology, College of Life Sciences, Zhongnan Hospital of Wuhan University, Wuhan University, Wuhan, China; ^5^ The National and Local Joint Engineering Laboratory of Animal Peptide Drug Development, College of Life Sciences, Hunan Normal University, Changsha, China

**Keywords:** ion channel, function, regulation mechanism, trafficking, pain, drug, therapeutic treatment

Ion channels allow ions to pass through cell membranes and are essential for a host of cell functions in many organs. Due to localization in primary sensory neurons and other key structures in pain processing, ion channels are regarded as a major class of drug targets for modulating pain sensation and controlling chronic pain. Among these channels, sodium, calcium, transient receptor potential (TRP), and PIEZO and purinergic P2X3 channels have been reported in the basic biology of senses and have great potential to combat chronic pain. The 2021 Nobel Prize (Ledford and Callaway, 2021) was awarded to two scientists who uncovered the mechanisms for how the nervous system senses pressure *via* Piezo1 and Piezo2 ion channels (Coste et al., 2010); and senses temperature by another ion channel called TRPV1 (Caterina et al., 1997; McKemy et al., 2002). Piezo2 also mediates inflammation- and nerve injury-induced sensitized mechanical pain, suggesting that targeting Piezo2 might be an effective strategy for treating mechanical allodynia (Murthy et al., 2018). The TRPV1 receptor has also been reported to play the role of analgesia in chronic neuropathic pain (Davis et al., 2000; Caterina and Julius, 2001; Proudfoot et al., 2006). Clinical studies of P2X3-containing receptors have shown promise in treatments for bladder pain and pain associated with osteoarthritis (Abdulqawi et al., 2015; Smith et al., 2020). Further investigation of the mechanisms of ion channels that are related to nociception could be key in developing a new class of therapeutics for many diseases, including pain.

Among the Food and Drug Administration (FDA)-approved drugs, 18% of drugs targeting human receptors target ion channels (Santos et al., 2017). For example, Ziconotide is licensed for severe, intractable, and chronic non-malignant cancer-related pain. In the European Union, dermal patches containing 8% capsaicin (NGX-4010) are currently approved for treating various forms of peripheral neuropathic pain (Peppin et al., 2011). Pregabalin introduced by Pfizer and sold under the brand name Lyrica, was approved by FDA in 2004 for the treatment of neuropathic pain associated with diabetic peripheral neuropathy and postherpetic neuralgia. It acts by targeting the voltage-dependent Ca^*2*+^ channel (Hundehege et al., 2018).

The Research Topic includes five original research papers from prominent researchers in the field and provides readers of the journal with recent results in the area of mechanisms of ion channels in pain, as well as new strategies for treatment.

The voltage-gated Na (+) channel Nav1.8, which is mainly expressed in dorsal root ganglion (DRG) neurons and sensory fibers (Akopian et al., 1996), contributes to the majority of the Na (+) current that underlies the depolarizing phase of action potentials, leading to its attracting interest as a potential target for pain. Gain-of-function mutations of the SCN10A gene (encoding the Nav1.8 channel), including an amino acid substitution from glycine to serine at position 1,662 (SCN10A G1662S), that produce DRG neuronal excitability have been found in 3% of patients with small fiber neuropathy (SFN) (Han et al., 2014). To date, the role of SCN10A G1662S in pain sensation has been relatively unstudied. Chidiac et al., were the first to generate a G1662S mutation mouse model for pain behavioral studies that evaluated mechanical sensitivity, proprioception and heat/cold sensitivity. The mutant animals manifested the pain features of the SFN patients carrying the SCN10A G1662S mutation, such as sensitive skin and abnormal thresholds for warm and cold stimuli as revealed by quantitative sensory testing. Moreover, male mice were more sensitive to exogenous heat than female mice.

In constrast, Nav1.7, another voltage-gated Na (+) channel alpha-subunit, was found to play less of a role in setting mechanical and thermal pain thresholds in comparison with Nav1.8 (Nassar et al., 2004). Kwon et al., investigated the relationship between neuronal activity and Nav1.7 expression in the trigeminal ganglion (TG) following allyl isothiocyanate-induced pulpitis. Strikingly, the nociceptive signal processing in the TG following pulp inflammation could be modulated by the inhibition of the Nav1.7 channel. In the future, it would be exciting to find specific Nav1.7 blockers for treating patients with inflammatory pain.

GABAergic progenitor cell transplants may contribute to the restoration of the spinal GABAergic inhibitory system, and provide long-lasting pain relief (Llewellyn-Smith et al., 2018). However, more studies are needed for the better understanding of chronic pain–associated plasticity within the peripheral GABAergic system. Wang et al. performed a systematic investigation of plastic changes of the GABA related proteins in the DRG in the process of neuropathic pain development. They found that most GABA_A_ channel subunits and GABA producing enzymes (the glutamate decarboxylase isoforms GAD65 and GAD67) were downregulated following chronic constriction injuries (CCIs) of the sciatic nerve, except the α5 GABA_A_ subunit, which was consistently upregulated under the condition of neuropathic pain. Furthermore, the knock-down of α5 GABA_A_ subunit *in vivo* moderately alleviated neuropathic hyperalgesia, suggesting a novel therapeutic target for pain relief.

The TWIK-related potassium channels, TREK-1 and TREK-2, are highly expressed in sensory neurons and contribute to the resting membrane potential of sensory neurons. Their inhibition could increase their excitability, resulting in the sensation of pain. Cunningham et al. discovered that the FDA approved drug Treprostinil, which has been used for the treatment of pulmonary arterial hypertension, is a potent antagonist of human TREK-1 and TREK-2 channels. They also found that the inhibitory effect of Treprostinil is reduced when channel activity is enhanced either by a gain of function mutation of TREK-1 (Y284A) or by BL-1249, a pharmacological activator of TREK channels. The study suggests that the topical application of specific activators of TREK channels may provide a safe therapeutic strategy for overcoming the excruciating pain experienced by patients receiving Treprostinil injections.

Gallic acid (3,4,5-trihydroxybenzoic acid) is a plant-derived compound with many drug effects, such as analgesia, anti-tumour and anti-inflammatory. However, the underlying mechanism of gallic acid in analgesia remains little studied. Yang et al. demonstrated that gallic acid could alleviate neuropathic pain behaviors in rats with CCIs. The expression level of the P2X7 receptor in CCI rats was increased after a 1-week administration of gallic acid, however, TNF-α, NF-κB and phosphorylated-STAT3 on satellite glial cells (SGCs) in the DRG were reduced. Furthermore, gallic acid significantly decreased the co-expression of the P2X7 receptor and glial fibrillary acidic protein in the DRG and the ATP-activated current in HEK293 cells with transient expression of the P2X7 receptor.

Overall, this Research Topic summarizes important findings related to ion channels, their mechanisms of regulation, novel compounds targeting ion channels and new strategies of treatment. The Research Topic provides new possibilities for exploration and research on ion channels and their potential for pain treatment.
